# “This is not my decision; I have no alternative”. Perceptions and experiences of marriage age and family planning among Syrian women and men: a primary care study

**DOI:** 10.1017/S1463423621000220

**Published:** 2021-06-07

**Authors:** Pinar Döner, Kadriye Şahin

**Affiliations:** 1 Family Medicine Department, Hatay Mustafa Kemal University Faculty of Medicine, Hatay, Turkey; 2 Social Anthropology Department, Faculty of Arts and Sciences, Hatay Mustafa Kemal University, Hatay, Turkey

**Keywords:** family physician, primary health care, refugee, reproductive health family planning, Syrian migration, women health

## Abstract

**Purpose:**

Reproductive health includes the capability to reproduce and the freedom to decide. In this context, both women and men have rights. In this study, it is aimed to reveal the obstacles in using these rights and to describe perceptions on marriage and family planning (FP) of Syrian women and men and to increase awareness for developing new policies on the Primary Health Care.

**Methods:**

The study was conducted using qualitative method, consisting of in-depth interviews with 54 participants; 43 women and 11 men who had to emigrate from varied regions of Syria at different times since 2011. Syrian women living in Hatay, in the south of Turkey were identified from Primary Health Care Center. Most of the Syrian women had given birth to the first two children before the age of 20 years. The interviewees were selected by purposive and snowball sampling.

**Results:**

The result was examined under seven headings: knowledge about FP and contraceptive methods, hesitation about contraceptive methods, emotional pressure of family and fear of maintaining marriage, embarrassing of talking about sexuality and contraception, the effects of belief and culture on contraception, psychological reflections of war, and changes in the perception of health during the process of immigration. The most significant factors affecting the approaches to FP and contraceptive methods of the women in this study were determined to be education, traditions, economic status, and religious beliefs. The most important factors affecting participants’ FP and contraceptive method approaches are education, cultural beliefs, economic status, and religious beliefs.

**Conclusions:**

The primary healthcare centers are at a very strategical point for offering FP services to help address patients’ unmet contraceptive needs and improve pregnancy outcomes.

More attention should be paid to social determinants that influence the access to reproductive health. Moreover, efforts can be done to address gender inequality that intercept FP. The most important strategy for primary health systems to follow the gender barriers that hinder access to FP services and men are empowered to share responsibility for FP.

## Introduction

Immigration is an important phenomenon that has been observed in every period throughout human history and the results of this have constituted a field of study for many disciplines (Koçak and Terzi, 2012; Şen, 2014). In recent years, millions of people have been on the move as immigrants, asylum seekers, or refugees. Today, there are about one billion immigrants, and the immigrant population is one-seventh of the global population (WHO, 2019). According to the International Migration Report 2015, the number of international immigrants has been rapidly increasing (McAuliffe and Ruhs, 2018). Although the number of international immigrants worldwide was 173 million in 2000, it reached 222 million in 2010 and 244 million in 2015 (McAuliffe and Ruhs, 2018). Migrations occur as a result of global problems such as economic crises, terrorism, civil wars, climate change, and ecological problems that negatively affect people and reduce the welfare of nations (Sirkeci and Cohen, 2016; Tirado, 2020).

Turkey is one of the countries where there has been an increase in the number of international immigrants. A large part of the migration is made up of Syrian immigrants, who left their country after the onset of civil war. The internal conflicts that began in Syria in 2011 caused thousands of Syrians to leave their country, with 5 million and 500 thousand Syrians immigrating to different countries in the search for a safe place (Samari, 2017).

According to the United Nations Office for Refugees, the conflict in Syria is seen as a civil war and has since been accepted as a civil war, leading to 5.4 million refugees in neighboring countries and 6.6 million internally displaced in Syria (UNHCR, 2019a; 2019b). However, especially the implementation of an open-door policy and temporary protection has increased the size of the migration to Turkey (Erdogan, 2015). According to a field studies report carried out in Turkey in 2017, the total number of Syrians in Turkey had reached 3,020,654 as 247,000 living in camps and 2,774,000 living outside camps. The Syrians living in Turkey were initially recorded as “guest” and then as “persons who were taken under temporary protection” (AFAD, 2019).

Hatay was one of the most affected places in the first stage of the emigration from Syria and remains so. At the same time, Hatay is a city where there are people living who have many relatives living in Syria, where there was intensive border trade before the war and where Arabic is widely spoken in addition to Turkish. According to the 2017 AFAD report, there were 357,954 Syrians living outside camps settled in the city of Hatay, which was the second largest Syrian population living outside camps (AFAD, 2019).

One of the healthcare services most needed by immigrants/refugees is women’s healthcare services. Women, elders, and children are the most affected by migration worldwide. The gender role of women, poverty and deprivation, lack of health insurance, or insufficient information prevent women from adequately benefiting from healthcare services. Therefore, immigrant/refugee women are more and more frequently confronted with problems related to women and reproductive health than locally resident women. The most important problems of these are the lack of family planning (FP) services, unwanted pregnancies, and abortion and birth complications. It has been reported by the World Health Organization (WHO) that 214 million women living in developing countries do not use modern contraceptive methods (WHO, 2019). In addition, family physicians have an important role at these services.

The aim of this study was to reveal experiences and perceptions of marriage age and FP among Syrian women and men. Besides this, the second aim was to reveal the changes of perceptions after settlement and to determine the needs and barriers of FP.

## Methods

This study was prepared using a qualitative research method. Qualitative methods are frequently used in health researches to identify complexities of life experiences (Liamputtong and Ezzy, 2005).

Medical anthropology conducts qualitative studies with a focusing on culture that how beliefs and traditions affect perceptions and practices of health. On the other hand, Family Medicine frequently encounters patients from different cultures and health problems while providing primary health care services. The health beliefs and behaviors of these patients are products of their own culture, and to benefit from medical anthropology need to understand it (Yaphe, 2014). Anthropology uses the qualitative research method by directly communicating with the participants about a situation and makes an important contribution to understanding this situation (Harper, 2006). Family Medicine deals with biopsychosocial and cultural values with a holistic approach (WONCA, 2005). For this reason, this study was conducted using qualitative research method, with the common approaches of Family Medicine and Anthropology.

Ethics approval was granted by the Hatay Mustafa Kemal University Social Sciences Ethics Committee, and permission for the field research was obtained from Hatay Governorship.

## Sample selection, inclusion criteria, and data collection

Both women and men were included in the study. The population of study comprised participants aged ≥ 18 years that had come from Syria and were living in Antakya districts in Hatay outside the camps. It is considered that the age range of the participants will affect the perceptions, attitudes, and behaviors of FP; therefore, the age groups of participants were categorized as 18–25 years, 26–35 years, 36–49 years, and ≥50 (Table 1). Syrian women and men were identified from Primary Health Care Center records. As inclusion criteria; all participants were born in Syria and had migrated to Turkey after Syrian civil war. The participants had migrated from different parts of Syria (Idlib, Aleppo, Latakia, Hama, Homs, and Damascus) at various times, and conflicts have occurred at different times in these parts. It is thought that this situation may affect the opinions of the participants about FP as it changes the year of immigration. For this reason, the city information and migration years of the participants are presented in Table 1.

**Table 1.** The distribution of participants by the year of immigration and the regions from which they emigrated

	Women participants									Men participants								
	Immigration years									Immigration years								
	2011	2012	2013	2014	2015	2016	2017	2018	Total	2011	2012	2013	2014	2015	2016	2017	2018	Total
Aleppo	2	2	1	3	–	–	2	1	11	–	–	1	–	1	–	–	–	2
Damascus	–	–	–	1	1	1	–	–	3	–	1	–	–	–	–	–	–	1
Hama	–	3	1	–	–	2	–	–	6	–	–	–	–	–	1	–	–	1
Latakia	–	–	2	1	2	3	2	–	10	–	1	1	–	–	–	–	–	2
Idlib	–	–	2	1	2	3	2	–	10	1	–	–	–	–	–	1	1	3
Homs	–	–	–	1	1	1	–	–	3	–	–	–	–	1	1	–	–	2
Total participants	2	5	6	7	6	10	6	1	43	1	2	2	0	2	2	1	1	11

Due to the fact that the opinions of both women and men are important and effective in FP decisions, it is important to have both genders in the sample of the study. Gender roles, patriarchy, religion, economy, and as tradition variables cause differences in women and men approach to FP. However, the main cause of the number of participants is less in man than women is that men do not want to talk about FP. In addition, some men expressed it as “shame” to talk about it.

The interviewees were selected by purposive sampling and snowball sample was also used to increase the possibility of different perspectives.

## Data collection

The interviews were conducted with verbal and written informed consent. Then, audio recordings were obtained from the participants with their permission before starting the interview.

In this study, in-depth interview was performed in Primary Health Care Center. The interview guideline was prepared both in Arabic and in Turkish (Box 1). The interviews were performed from 5 June to 31 October 2019. After interviewing with 43 Syrian women and 11 Syrian men, it was decided that the data were saturated and data collection was terminated. This was in line with the estimation of Morse, who estimated that 30–35 interviews provide data saturation (Morse, 2000). The analyses ran from November 2019 to January 2020.

Box 1. Interview Guidelines

What do you think about family planning? What are the contraceptive methods/Family planning?

How was family planning in the country you came from? What are your observations about family planning practice in the country you are in now? Do you have any information about the contraceptive methods given for free? Do you know how you can achieve these methods?

At what age do women usually get married in the country you come from? How old did you get married?

From whom, when, and how did you learn family planning?

Is there any information you hear or fear negative about contraceptive methods?

Can you talk about family planning among women? Do you explain and consult each other? What are you talking about?

Are you talking with your partner about family planning? What do you speak if you speak? If you are not talking, can you explain why?

How do you decide if you are using a contraceptive method? Would you decide with your partner? If you are not making a decision together, can you explain why?

Were you able to get information about contraceptive methods at school or in health centers before getting married? How was the application in the country you came from?

Are you talking with your partner about how many children you want to have in total? How do you determine the number of children you want to have?

How would you like family planning to be? What is the number of children in your ideal? How do you determine this? What are your thoughts that affect this?

How long do you think it takes to get pregnant after the last pregnancy? How should the frequency of birth be for you?

How is it met when you have few children in country you came from?

Is child gender important in the country you come from? Does the society want to have a boy or a girl more?

Is there a thought of giving birth until the number of girls or boys reaches a certain number in the country you come from?

### In-depth interview, interpreters, voluntary participation form, and participant form

Interpretation, which constitutes the theoretical basis of qualitative research, “involves the elaboration of representations such as in-depth interviews, participatory observation and life stories, with a relatively small number of individuals instead of large sample groups” (Kümbetoğlu, 2008).

Both interpreters could speak Arabic and Turkish, and the Syrian interpreter could also read and write Arabic. They were consulted during the interview and were informed in detail about the interview technique. Pilot interviews were conducted with interpreters by providing training on the interview technique used in the study.

The emotional state of the participants, body movements, and facial expressions were recorded during the in-depth interviews. In some cases, the interpreter was asked what the body movements mean and sometimes the women participants were embarrassed, their eyes filled with tears, and they lowered their voice related to the sensitivity of the subject. As a result of the pilot study, it was determined that FP is a sensitive issue for all Syrian participants. Before starting the study, there was a concern about whether the communication problem and the sensitive nature of the questions might prevent answers being obtained or whether the correct answers could be obtained. Therefore, before starting the interview each participant was informed that their names would not be disclosed. “The precondition for each successful qualitative interview is to establish a trust relationship between the *interviewer* and the *narrator*” (Van Liempt and Bilger, 2018), thus, questions were answered clearly, and interviews were initiated after the participants’ concerns about the study were resolved. Moreover, to communicate with women more easily and provide detailed information, the interviews were conducted by a woman interpreter with her positive, understanding, and explanatory behavior. Thus, worry about not received detailed and accurate information that is considered at the beginning of the study was also disappeared. In addition, despite the efforts of the translators and researchers to communicate in the interviews with the men, the information provided by the men remained limited. Although men were interviewed through a male interpreter, the fact that the person conducting the research and taking notes was a female caused men to sometimes avoid eye contact and act hesitantly.

A voluntary participation form was used in the study. The voluntary participation form contains information that participants shall participate in the study on a voluntary basis, that the data obtained from the interview shall be used only for scientific research and that the data shall not be shared. The interview form was prepared both in Arabic and in Turkish. Before the interview, a written informed consent form was also read to the participant verbally, and the participant was informed that she/he did not have to answer questions that she/he did not want to and could switch to other questions or terminate the interview if she/he wanted.

The participant form consisted of two parts. The first part comprised questions to obtain demographic information, including age, gender, marital status, education level, number of children, the place they lived in Syria, how long they have lived in Turkey, and how many people they are living with. The second part of the form comprised semistructured questions. A semistructured questionnaire with open-ended questions, which was priorly pilot tested to sort out any problems in understanding, was conducted. In addition, three pilot interviews were conducted with interpreters and some of the prepared questions were rewritten to be clearer. Participants were blinded to the purpose of the study. There was no time constraint for interviews. The interview time ranged from 30 to 70 min. All interviews were audio recorded and then transcribed verbatim for analysis.

### Data analysis

Data were analyzed using thematic analysis (Creswell and Poth, 2016). The interviews were analyzed following each interview. Assessment of each interview provided us new ideas. Different ideas led us to review the interview process dynamically and continuously (Ritchie and Spencer, 2002). The interviewing process was continued until no new data gained. Saturation of data was obtained after 43 interviews with women and 11 interviews with men.

The transcribed interviews were open-coded separately. The codes were grouped and analyzed for divergence and convergence. This process of divergence continued until no new codes and categories emerged. The codes were grouped into themes (Patton, 2002).

The themes identified during the analysis process were drawn from participants’ experiences and feelings. All inconsistencies were discussed, and the consensuses on the final concepts were reached. Transcripts were reviewed and the responses were categorized based on similarity. Then, reviewers discussed their findings. Reviewers had a consensus and the final findings were reviewed by two physicians who did not participate in the study; for credibility based on their own experiences.

Participants’ responses were quantified to enable how often participants emphasized same opinion. The numbers of women patients expressing a theme were grouped by quantifiers as follows: ‘a few’ 0–5 persons; ‘some’ 6–19 persons; ‘many’ 20–34 persons; and ‘most’ 35–43 persons.

## Results

The distribution of 43 women and 11 men participants was shown in Table 1 by the year of immigration and the region from which they emigrated.

It is considered that the age range of the participants will affect the perceptions, attitudes, and behaviors of FP; therefore, the age groups of participants were categorized as 18–25 years, 26–35 years, 36–49 years, and ≥50. When the educational status was assessed, 16 of the female participants were university graduates, 26 primary and high-school graduates, and one was illiterate. On the other hand, seven of the men were university graduates, three high-school graduates, and one primary school graduate (Table 2).

**Table 2.** The distribution of participants according to the gender, age groups, and educational status

	Women participants					Men participants				
	Age groups					Age groups				
	18–25	26–35	36–49	≤50	Total	18–25	26–35	36–49	≤50	Total
Illiterate	–	–	–	1	1	–	–	–	–	–
Primary	3	2	4	5	14	–	1	–	–	1
Highschool	4	5	3	–	12	–	1	–	2	3
University	4	9	2	1	16	–	4	2	1	7
Total participants	11	16	9	7	43	–	6	2	3	11

According to the data obtained from the interviews, the FP information of the participants varies according to the age and education. It has been observed that most of the participants aged 50 and above who have spent their reproductive ages in Syria have very little knowledge about FP, besides, they do not apply FP and even do not view it positively. Although most women of reproductive age between the ages of 18–25 and 26–35 have little knowledge about FP methods, it has been observed that they want to learn it and make researches on this subject in various sources. In particular, it was determined that all of the university graduate participants wanted to learn the methods and use them, and even some of them decided to implement FP by talking with their spouses.

Most of all female participants stated that having too many children negatively affects their lives and this situation is not caused by their own choices, but because of the pressure of their husbands and society. In fact, most of the women stated that they advised their children to marry at a late age, to have fewer children, and to implement FP.

It was determined in the interviews that the male participants who are university graduates have more information about FP. On the other hand, it was observed that university graduates also wanted to have a large number of children, especially boys, like other participants.

All participants immigrated to Turkey between 2011 and 2018 (Table 1). After migration, it has been determined that the knowledge of many women about FP increased via Primary Care Centers, with the time they spent in Turkey. In addition, it was also seen that they want to get more information about the FP implementations in Turkey and given a free. Although the vast majority of the participants came from cities such as Aleppo, Latakia, Hama, Idlib, and others which are closer to the border of Turkey, there is no significant difference in approachment to family planning among the participants.

The distribution of married women participants according to the marriage age, first pregnancy age, and educational status is shown in Table 3. Twenty-six of women had been married under the age of 18 years. The educational status of 13 women whose marriage age is under 18 was primary among all married women (Table 3). The distribution of married participants according to the gender, age groups, and number of children is shown in Table 4.

**Table 3.** The distribution of married women participants according to the marriage age, first pregnancy age, and educational status

	Marriage age		First pregnancy age	
	>18	≤18	>18	≤18
Illiterate	1	–	1	–
Primary	13	–	12	1
Highschool	9	1	3	7
University	3	9	3	9
Total participants	26	10	19	17

**Table 4.** The distribution of married participants according to the gender, age groups, and number of children

	Women participants					Men participants				
	Age groups					Age groups				
	18–25	26–35	36–49	≤50	Total	18–25	26–35	36–49	≤50	Total
Total number of children	3	37	48	33	121	–	14	7	13	34
Total number of girls	2	16	18	13	49	–	9	4	9	22
Total number of boys	1	21	30	20	72	–	5	3	4	12
Number of children born in Turkey	2	8	3	–	13	–	8	3	–	11
Current pregnancy status	1	3	–	–	4	–	–	–	–	–
Total participants	4	16	9	7	36	–	7	2	2	11

The analysis revealed seven main themes: (i) knowledge about FP and contraceptive methods, (ii) hesitation about contraceptive methods, (iii) emotional pressure of husband, family, and social environment and fear of maintaining marriage, (iv) embarrassing of talking about sexuality and contraception, (v) the effects of belief and culture on contraception, (vi) psychological reflections of war, and (vii) changes in the perception of health during the process of immigration. These themes provide the cultural context for understanding the attitudes and barriers regarding FP among Syrian women.

### Knowledge about FP and contraceptive methods

In the interviews we conducted on FP and accessibility to the contraceptive methods, most of participants did not know exactly what FP was and some of them had never heard of it. In addition, most of them had not used most of the contraceptive methods. There were also a few participants who did not feel the need to learn what FP means until the desired number of children is reached. Few of the participants stated that they did not know how to access the methods.

“*I didn’t know about FP and methods. My family say that when a child is born, it comes by chance.*” (21 years old woman, high school, Aleppo, 2012)

“*We usually use the withdrawal method. And sometimes we use condoms, nothing else.*” (20 years old woman, primary school, Idlib, 2017).

“*I don’t know about FP but I want to learn because I will get married soon and I will also need this information.*” (21 years old woman, high school, Aleppo, 2012).

“*I heard FP but I don’t want, I did not want my wife to use pills. We tried to protect ourselves, but my wife became pregnant and had 8 children. God gave to us…*” (50 years old man, university, Idlib, 2018)

It is observed that using the internet, which is the easiest way to access information, increases the knowledge of FP, which is considered sensitive. Because, most of the participants stated that they do not have information about FP or they cannot talk about this issue with everyone in their country where they come from: *“FP…I searched this from internet and learned some information about methods. But I don’t know how I can take those methods.”* (30 years old man, high school, Homs, 2016).

Social imposition, husband, and family pressure determine women’s perspective on FP:

“*My husband and his family want more children, probably we will have five children or more. I’m pregnant now, maybe I will learn after five children but I don’t need any method now, so I didn’t ask anyone about methods*” (18 years old woman primary school, Hama, 2016).

### Hesitation about contraceptive methods

Some of the participants stated that they were informed about the side effects of some of the contraceptive methods and that the information they heard from their social environment affected their preferences. Especially with regard to oral contraceptive use, some of the participants stated that they did not prefer this method because of fear of misuse or incomplete use.

“*They say pills damage hormones and cause cysts in ovaries, so I don’t use them.*” (35 years old woman, primary school, Homs, 2016).

“*I heard about pills, but they say pills cause headache and anger.*” (42 years old woman, primary school, Aleppo, 2013).

“*I’ll have an intrauterine device because I cannot follow the shapes on the pill, and I may be confused. I might also forget to take it day by day.*” (17 years old woman, primary school dropout, Idlib, 2017).

“*I did not want my wife to use pills*” (50 years old man, university, Idlib, 2018)

### Emotional pressure of husband, family, and social environment, and fear of maintaining marriage

There is an emotional pressure on women who have few children to have more. Most of the women emphasized their husband, family, or social environment insist to have more children. In addition, there is a high expectation of gender of child. Almost all of the participants stated that the most important reason preventing FP is the desire of a boy. Fear of maintaining marriage is one of the most important and effective barriers to FP. Some participants stated that polygamous marriages (second, third, or fourth wives called ‘kuma’) may occur in Syria if they fail to have many children or especially to give birth to boys. There were participants who stated that this situation caused women to give birth even if they did not want to.

“*Especially mothers with daughters are under pressure to go on having children until they have a son.*” (20 years old woman, primary school, Idlib, 2017).

“*In Syria they love sons, they want more sons. I think the number of children is enough but my husband would want more.*” (31 years old woman, primary school, Hama, 2016).

“*In Syria people want more children, they say you have to more children to maintain the continuation of the family.*” (39 years old man, university, Hama, 2016)

“*If the wife doesn’t accept to give another birth for son, the man can go and get another wife to have a son. They attach more importance to boys. In Syria most of the families with 6–7 children, people don’t have few children.*” (48 years old woman, married, primary school, Aleppo, 2013).

### Embarrassing of talking about sexuality and contraception

Most of the women have low knowledge about contraception before their first pregnancy and they had not talk to their mother about sexuality, FP, and contraceptive methods because it was considered embarrassing to talk about these issues and some of them asked to their friends or searched from internet. Most of the men said that they had learned some information from their friends or relatives. Men gave very short answers about this subject.

“*My mother didn’t talk to me about sexuality, my friends gave some information. After marriage my husband tested me, a week after marriage he told me; If I knew anything, he would have a negative opinion of me. Then I understood that talking about sexuality in our culture is a big shame, even connecting with whether you are virtuous. In fact I don’t think like that, if you don’t know something about sexuality and FP, you feel big fear and shame.*” (32 years old woman, highschool, Latakia, 2014).

### The effects of belief and culture on marriage age and contraception

One of the reasons that prevent FP is the perception and attitudes related to beliefs. The belief that the number of children is under the control of Allah (i.e., God) and that spouses talk about it creates one of the controls on the fertility of women, and it was stated by the participants that this idea creates an obstacle to FP. Besides, in their culture sons are more important because they believe that boys carry surname and support the family. So, most of the families continue to give birth until they have sons. The participants emphasized that they do not usually talk with their husbands about how many children they will have, and that the number of children is considered to be completed when the general acceptance of society is reached. Belief and culture affect the number of children together. In addition, belief and culture affect the marriage age, most of the women emphasized that most of the girls had to marry at 16–17 years age. Most of the men emphasized that girls had no enough safekeeping if they had not married. In addition, if they had not married, they would not have enough financial support to live.

*“We don’t talk to my wife about the number of children, Allah, our creator gives us these children and they bring their luck with them.”* (35 years old man, primary school, Aleppo, 2013).

“*In our culture, they absolutely want sons. Even if you have 6 daughters, you give birth to the seventh until you have a son. A son is better in Syria because they think he carries his father’s surname and he will support the family.*” (27 years old woman, primary school, Idlib, 2013).

This 17-year-old participant emphasized with a sad and desperate expression that her choices are made by culture while explaining her own situation during the interview. It was observed that the thought that had no say in the decision affected this woman, who was still at a very young age, emotionally:

“*I’ve never told to my husband about how many kids we’re going to have. In our culture, they say children are the most valuable jewelry in the world. They definitely want boys. I think we can have 5 children, generally families have 5–6 or 7 children. This is not my choice, I have no alternative…*” (17 years old woman, primary school, Idlib, 2017).

It is understood that early marriages leave deep scars on the psychology of women. During the interview, the feeling of fear experienced by a woman who got married at an early age and her sadness about it were also understood from her body language:

“*The age of marriage in Syria varies between 13, 14, 15 and 20 years. I got married when I was 13 and I was very young; I was so scared.*” (27 years old woman, middle school, 2013, Homs)

Another participant who indicated that 25 is the “ideal age” for marriage because early marriage adversely affects health stated that she will let her daugher marry at the age of 25 and that early marriage is ignorance:

“*Because of bad health conditions and the idea of early marriage, she can’t handle with marriage. Now I have a 17-year-old single daughter and I don’t think I’ll let her marry until she is 25, she must be mature enough to carry the responsibility of a marriage. It is completely ignorance. When the girl is older, she will be more knowledgable about issues such as FP and sexuality*” (54 years old woman, middle school, 2015, Homs)

### Changes in the perception of health after settlement and psychological reflections of war

Unlike the opinions of other participants, while talking about the number of children that families want, some of the participants stated that they consider economic conditions as the reason why people want fewer children in Turkey compared to Syria. Besides they thought marriage law and FP education are better in Turkey, some of them had awareness regarding this subject. Thought of compensating is one of the most important reasons that affect FP. There were also those who thought that the desire to have many children was caused by war, to compensate for the death of too many people. While talking about this topic, it was observed that one of the participants was very sad and emotionally worn out:

“*Some of us give birth to a lot of children because of war, many people died, they might want to compensate, and there is no planning.*” (38 years old woman, university graduate, Latakia, 2014).

As a result of this interview, it is understood that the decrease in the population due to traumatic deaths and devastating losses has a significant effect on people’s psychology. Considering the situations that increase the desire to have more children, it is seen that psychological traumas and the desire to survive, the effort to survive in life, the desire to remain strong against difficulties as a large family are effective. This actually creates a psychological burden for both women and men.

“*We want more children, I love big family, many young men had died during war, so I want big family again.*” (34 years old man, university graduate, Latakia, 2013).

“*After coming to Turkey, many of my friends have had the idea of having fewer children from diseases of poverty.*” (20 years old woman, primary school, Idlib, 2017).

It is clearly seen that a lot of information about birth control methods has been learned by both men and women after migration:

“*In Turkey, doctor gave me information about methods at the refugee health center and told me I could take free condoms, I hadn’t known that these methods were safe and free.*” (35 years old man, highschool, Homs, 2015)

“*I learned information at refugee center, then I told my husband what I learned and said that we must think our children’ future so we had to choose one of these methods.*” (30 years old woman, highschool, Damascus, 2017).

While the participant, who got married at the age of 15, was talking, it was observed that she felt regret about the age of marriage and faltered by looking down with sad eyes. She stated that they get better education for their daughters in Turkey, therefore, while she hoped for a good education and FP for his daughters, her voice was more vivid.

“*I wish got married after graduation, I got married at 15 years old woman. In Turkey girls are supported to complete their education. Most of the girls want to have a job and they want to work after education before marriage. I wish my daughters could complete their education and have a good job and then have children but fewer children.*” (42 years old woman, primary, Aleppo, 2013).

## Discussion

Migrant women face many socioeconomic, psychological, and physical problems regardless of country or culture. Syrian migrant women also need biopsychosocial support while trying to live in the countries where they have migrated. One of the issues that they need support is reproductive health in primary care. Many Syrian women face pregnancy-related complications and also mortality due to insufficient access to reproductive health services. Several studies have been conducted related to the use of FP services by Syrian women (Erdogan, 2015; Sirkeci and Cohen, 2016; Öztürk, 2017; Alan Dikmen *et al.*, 2019). Mother and baby mortality and morbidity can be prevented with accessible and acceptable FP services.

The current study can be considered important in respect of revealing how the perceptions of FP of women and men emigrating from Syria have been affected by the immigration and in terms of examining the level of knowledge of FP, the demands for FP, and the ability to access FP methods within the primary healthcare services.

In addition, the strengths of this study are: although, there are many quantitative studies related with only Syrian women, this is the first qualitative study in primary healthcare services of Turkey that includes interviews with both Syrian women and men. Also we need to understand their perceptions about “ideal marriage age” and FP. The participants are from different age groups and education levels. It is important that the participants consisted of women who had married at a child age, single women, and women above the age of 50 who married for many years, allowing us to create a large pool of qualitative data.

The vast majority of the participants in this study reported low knowledge of FP. The information that FP methods are free and that information can be obtained from the Primary Healthcare Centers and Refugee Healthcare Centers must be more widespread. In addition to seminars given to women presenting at the healthcare centers for various reasons, information can also be given on a one-to-one basis in their homes. Besides, it was understood that of those who had knowledge, there were barriers to implementation such as various fears, hesitations, and psychological pressure. The most frequently emphasized barriers were concerns about side effects, fear of loss of fertility, and exposure to polygamy because of FP. On this basis, a man is free to take more than one wife with the condition in Islamic religion that “there is equal distribution of material resources among wives and equal treatment of all wives,” men in the Syrian Arab Republic are free to take more than one wife and can legally marry four women. As polygamy is legally established and men are used to this situation, the fear of polygamy is reinforced in women. This fear causing women to give birth to more children is a significant reason preventing FP.

In a study by Dikmen *et al.*, the level of education was found to affect the status of using FP methods (Alan Dikmen *et al.*, 2019). In contrast in our study, it was determined that apart from knowing a few names of FP methods, those with a high level of education did not have sufficient knowledge about how to use the methods or side effects, and the withdrawal method was preferred by the majority. Culture and belief could shape women’s knowledge regarding sexual health and FP even if their education level is high.

The greatest barriers determined in this study to the use of FP methods, which are provided free of charge in Turkey, were fear of side effects, the belief that these methods would cause infertility, and that their husband would not want to use these methods. Similarly, a study in Lebanon-reported cost, and fear of distance and misuse, and discrimination as barriers to accesssing reproductive healthcare services (Cherri *et al.*, 2017). In a similar study in Jordan, uncertainty of the efficacy of the methods and fear of side effects were stated as the main barriers (USAID, 2018). Those women were concerned about side effects and frightened of forgetting to take pills were the reasons reported in another study in Lebanon (Kabakian-Khasholian *et al.*, 2017). West *et al*. conducted a study of Syrian women living in a refugee camp in Jordan and reported that the participants had concerns related to contraceptive methods (West *et al.*, 2017). The findings of the current study and those of previous studies indicate that even if these women know about FP methods, they can remain distanced from them because of fears and concerns.

As many participants received negative explanations about FP methods from their social environment, they preferred not to use these methods rather than obtain information from doctors or healthcare workers. In addition, a significant proportion of the women in the current study did not know that there were free methods and free consultation services or how to access these. Therefore, it is extremely important that immigrant women who present and are followed up for any reason at healthcare centers are educated on these subjects and that their fears and concerns are clarified.

In studies that have examined the high-birth rates of Syrian women in Turkey, reasons have been reported to be the desire of husbands or family elders for many children, the need for a labor force, raising someone to work the family land, the wish to protect the family lineage, and state support as a policy for many children. In addition, as the mother of a child, the status of a Syrian woman is increased in society. It has also been reported that because of the competition originating from polygamy, which is a traditional practice in Syrian society, the women try to keep their husbands close by having many children (Alan Dikmen *et al*., 2019). In a study conducted in Lebanon by Kabakian-Khasholian *et al*., it was emphasized that even if women wanted to have fewer children to be able to provide them with a better education, they were hesitant about this with their husbands because of the fear of polygamy (Kabakian-Khasholian *et al*., 2017). Similarly, in the current study, the majority of participants explained that if they did not have many children in Syria and especially if they had failed to produce a male child, there could be polygamy. In some patriarchal societies which are maintained through the male line, female children are seen as guests who will one day be married and go to another family. This was also seen to be a reason for Syrian women having many children even if they did not wish to.

It was emphasized by the vast majority of the current study participants that the desire for a male child became pressure, especially from the husband’s family and relatives. Only having a female child is never seen as enough and births are continued until there is a male, regardless of the number of births. The majority of Syrians are dominantly believed that the lineage continues with boys, as a result, men feel the responsibility to continue the lineage. This approach results in the desire to have many children to have boys in most of the participating men. This situation is the reflection of gender roles in the field of health. This desire for a male child, and this imposition on the woman especially by the surrounding relatives, is not a situation that shows much variation according to the level of education, and this can become a great psychological pressure. Therefore, knowledge of FP methods or having a high level of education remains insufficient for the implementation of FP methods. Thus, further steps on the subject of FP will only be able to be made with the education not only of women but also of their husbands. Several women in the current study stated that they needed permission from their husbands to use FP methods and generally the men did not wish to use protection. Even if the man approved the use of protection, they explained that the woman had to provide the means of protection against pregnancy. The majority of the women interviewed in our study stated that they thought protection without the knowledge of their husband was sinful behavior and as their religious beliefs suggested that families have many children they were also personally inspired by this belief. Therefore, education will not be sufficient for women only, education programs are essential for men. It may be useful to conduct interviews with men, first their thoughts and psychology could be understood, and then information could be shared interactively. Thus, it could be useful to invite and motivate them to change behavior. Informative studies can be made on the subject of societal gender and equality. Studies can be conducted to develop a cultural perception that would prevent societal gender inequality which emerges with different dynamics and which is reflected in the field of healthcare.

It was understood that many participants were embarrassed to respond to the issues of sexuality and FP as same as with their family and friends. Sexual life before marriage is considered a sin and is considered culturally shameful. Therefore, as in the study of Chi Watts *et al*., young people cannot talk about these issues with their families before marriage (Ngum Chi Watts *et al.*, 2015). This situation may cause young people to get wrong information from wrong sources. In our study, it was observed that the interviewers, who were ashamed of talking at first, became enthusiastic to speak and even to receive information by providing confidence and comfort. Thus, encouraging families to talk to their children during the trainings and speaking to the youth by providing confidence will increase the level of knowledge.

In contrast to this finding of our study, in a study conducted in Lebanon by Cherri *et al*., it was concluded that religion did not constitute a basic obstruction on this subject (Cherri *et al*., 2017). Moreover, in the same study in Lebanon, the majority of Syrian women believed that a couple living in Lebanon should have fewer children than was normal in Syria, and the main factors influencing this decision were education, economic status, the ability to meet the needs of the children, and the uncertainty of the costs of pregnancy and birth to which they would be exposed (Cherri *et al*., 2017). In our study, the women who could not implement FP for themselves emphasized that they had wanted to when their children got married to be able to provide a better quality of life for them. Women’ perceptions could change after settlement. They faced to new culture, new economic conditions, and new health policy. These new and different situations could be effective on their decision.

The unmarried participants in our study stated that they had no knowledge of FP and felt a need for that information, and that insufficient information was given in school. Those who are single will probably learn about FP through experience after marriage. Therefore, it is important to include singles in FP and sexuality training programs. The subjects could be expanded so that by explaining reproductive health and the incorrectly acquired information of indications and side effects of contraceptive methods, the lack of knowledge and fear could be reduced. It can also be considered that increasing the level of knowledge and learning how to access healthcare services would contribute greatly to reducing mother and infant mortality.

## Conclusion

The primary healthcare centers are at a very strategical point for offering FP services to help address patients’ unmet contraceptive needs and improve pregnancy outcomes. This study has revealed the vulnerability of primary care services for migrant women. The most significant factors affecting the approaches to FP and contraceptive methods in this study were determined to be education, traditionals, economic status, and religious beliefs. The greatest barriers to FP were seen to be the desire of the family, or especially the husband, to have many children, pressure on this subject from the family and social environment, the thought that it is necessary to have many children to maintain the marriage, religious beliefs, the desire for a male child and the thought that it is necessary to continue having children until a male is born, the insistence of family, husband, and society to have a male child, not using FP methods because of little or no knowledge or incorrect knowledge, and not having information about accessing FP methods and that they are free of charge. The most important strategy for primary health systems to follow the gender barriers that hinder access to FP services and men are empowered to share responsibility for FP.

## Key messages

- Health professionals often encounter unmet needs of the reproductive health of migrant women. There are many quantitative studies examining the knowledge and attitudes of migrants on family planning. But, these studies do not provide detailed information.
- Our qualitative detailed examination of the problems experienced by migrants will enable us to identify the deficiencies in migrant health management and give an idea to shape policies.
- More attention should be paid to social determinants that influence the access to reproductive health. Moreover, efforts can be done to address gender inequality that intercept family planning.
- The special needs of migrants should be taken into account and reproductive health trainings can be given more frequently to migrants and protective laws can be developed by the health authorities.
